# Antimicrobial Activity of Lipid Extracts of Echinoderms in the Nhatrang Bay (South China Sea)

**DOI:** 10.1134/S1607672922020119

**Published:** 2022-05-10

**Authors:** E. S. Obukhova, A. M. Rozhina, V. P. Voronin, P. Yu. Dgebuadze, S. A. Murzina

**Affiliations:** 1 Institute of Biology, Karelian Research Center, Russian Academy of Sciences, Petrozavodsk, Russia; 2 Petrozavodsk State University, Petrozavodsk, Russia; 3 Severtsov Institute of Ecology and Evolution, Russian Academy of Sciences, Moscow, Russia

**Keywords:** antimicrobial activity, nosocomial strains, lipids, fatty acids, tropical ecosystems

## Abstract

The obtained results on the study of the antimicrobial activity of lipid extracts of tissues of starfishes *Linckia laevigata* and *Culcita novaeguineae* and sea urchin *Diadema setosum* collected in the Nhatrang Bay (South China Sea) against nosocomial strains of *Klebsiella pneumoniae*, *Enterococcus faecium*, *Escherichia coli*, *Pseudomonas aeruginosa*, *Candida* sp., *Streptococcus pyogenes*, and *Staphylococcus aureus* are presented. The effect of the investigated extracts on Gram-positive, Gram-negative microorganisms, as well as yeast of the genus *Candida*, was determined. It was found that lipid extracts of echinoderms of the Nhatrang Bay exhibit the highest antimicrobial activity against the Gram-positive microorganisms, namely *Streptococcus pyogenes.*

The discovery of antibiotics heralded a new era in medicine. However, decades later, bacterial infections remain a global threat to public health due to the increasing antibiotic resistance of microorganisms [1, 2]. Therefore, it is necessary to search for new biologically active compounds with antimicrobial properties [3].

Along with the prospects of studying antimicrobial peptides, the mechanism of action of which is associated with the disturbance of the structure of the bacterial plasma membrane [4], substances of a lipid nature are currently considered as potential antimicrobial agents. In addition to the well-known importance of ω-3 polyunsaturated fatty acids (ω-3 PUFAs) for human nutrition and health, anti-inflammatory and antioxidant activity was established for this group of compounds [5]. The mechanisms of antibacterial properties of PUFAs are determined by their action on the cytoplasmic and outer membranes of bacterial cells and include the disruption of the electron transport chain, uncoupling of oxidative phosphorylation, inhibition of enzyme activity, disruption of nutrient transport, and peroxidation, eventually leading to the bacterial cell death [6].

Alternative and relatively safe sources of “marine” lipids promising in terms of their antibacterial activity can be echinoderms, which are widespread in tropical ecosystems, many of which are popular objects of aquaculture in the Indo-West Pacific region.

In this study, we investigated the antimicrobial activity of lipid extracts of tissues (liver and gonads) of starfishes *Linckia laevigata* (Linnaeus, 1758) and *Culcita novaeguineae* (Müller and Troschel, 1842), as well as the sea urchin *Diadema setosum* (Leske, 1778), which are abundant species in the Nha Trang Bay (South China Sea).

Total lipids from the tissues of the studied species of echinoderms *L. laevigata* and *C. novaeguineae* (liver, *n* = 15) and *D. setosum* (*n* = 15) were isolated according to Folch [7]. Individual lipid classes were analyzed by high-performance thin-layer chromatography (HPTLC), individual phospholipid fractions were analyzed by high performance liquid chromatography (HPLC), and complete fatty acid profiling of total lipids was performed by gas chromatography (GC) [8]. The scientific equipment of the Center for Collective Use of the Karelian Research Center of the Russian Academy of Sciences was used in the study. The obtained data on the composition of lipids and fatty acids of the studied tissues were compared with the results of antimicrobial activity. For this purpose, after removing the chloroform–methanol mixture, dimethyl sulfoxide (DMSO) was added to the lipid extracts at concentrations of 30, 50, and 90%. Different concentrations of DMSO were taken due to the fact that DMSO is an antimicrobial agent and is used in medical practice to treat infectious diseases at different concentrations depending on the type of infection. DMSO was added to the extracts to create an emulsion that allows the extracts to be added in a dropwise manner onto Petri dishes with microorganisms.

Screening of antimicrobial activity of lipid extracts of the studied echinoderm tissues was performed against nosocomial strains of *Klebsiella pneumoniae*, *Enterococcus faecium*, *Escherichia coli*, *Pseudomonas aeruginosa*, *Streptococcus pyogenes*, *Staphylococcus aureus*, *Candida* sp., which were isolated from a multidisciplinary hospital in Petrozavodsk. The studied material for culture isolation was wound discharge (*E. coli*, *E. faecalis*), urine (*Str. pyogenes*, *Ps. aeruginosa*), sputum (*Kl. pneumoniae*, *Candida* sp., *S. aureus*). For all isolated microorganisms, sensitivity to antimicrobial drugs was previously determined by the standard methods (Table 1).

**Table 1.** Antibiotic sensitivity of microorganisms

Microorganism	Antibiotic susceptibility profile	Antibiotic resistance profile
*E. coli*	FOX, C, SAM, CZA	CRO, CTX, CPM, CIP, CFX, CXM, AP, PRL, CAZ, ATM, TS, AC
*Kl. pneumoniae*	MEM, IMI, CZA	GM, AK, CRO, CTX, CPM, CIP, FOX, CFX, CXM, AP, PRL, C, CAZ, ATM, TS, AC, SAM
*Candida* sp.	FCN, AMB, KCA, MCL	
*Ps. aeruginosa*		CPM, CAZ, CZA, IMI, MEM, ATM, CIP, AK
*S. aureus*	OX, LZD, FOX, CIP, AK, TS	PG, C
*Str. pyogenes*	PG, VA, TS, LZD	C, E
*E. faecalis*	AP, VA, IMI	LZD

AMB—Amphotericin B, KCA—Ketoconazole, MCL—Miconazole, GM—Gentamicin, MEM—Meropenem, IMI—Imipenem, AK—Amikacin, CRO—Ceftriaxone, CTX—Cefotaxime, CPM—Cefepime, CIP—Ciprofloxacin, FOX—Cefoxitin, CFX—Cefalexin, CXM—Cefuroxime, AP—Ampicillin, PRL—Piperacillin, C—Chloramphenicol, CAZ—Ceftazidime, ATM—Aztreonam, TS—Trimethoprim-sulfamethoxazole, AC—Amoxicillin-clavulanic acid, SAM—Ampicillin-sulbactam, CZA—Ceftazidime-avibactam, LZD—Linezolid, OX—Oxacillin, PG—Benzylpenicillin, FCN—Fluconazole, VA—Vancomycin, E—Erythromycin.

To prepare the inoculum, daily cultures of bacteria grown on a dense nutrient medium were used, which were suspended in a sterile saline solution to obtain a microbial suspension with a density of 0.5 according to the McFarland standard. The bacterial suspension was seeded with a sterile cotton swab on Mueller–Hinton agar.

The Petri dish inoculated with microorganisms was divided into six sectors; 50 μL of lipid extracts were added to five sectors, which were previously dissolved in DMSO of 30, 50, or 90% concentration. The 6th sector, to which DMSO was added at 30, 50, or 90% concentrations, served as a control. The cultures were incubated at 37°C for 24 h, and *Candida* sp. was incubated for 48 h.

The growth retardation zone was determined visually according to the following scale: “–” no effect, growth retardation zone is absent; “+” bacteriostatic effect, growth inhibition is observed; “++” incomplete bactericidal effect, growth inhibition is observed, single colonies are visualized; “+++” complete bactericidal effect, complete growth inhibition. Figures 1 and 2 show the results of a qualitative experiment based on the principle of the presence/absence of the bactericidal effect. Each sample was applied in triplicate, the resulting seeding result was evaluated on the “scale of bacteriostatic action” in comparison with the control sample and averaged. For each microorganism strain, a frequency analysis was performed to identify the maximum antimicrobial activity at different concentrations of DMSO in echinoderm lipid extracts.

**Fig. 1.**
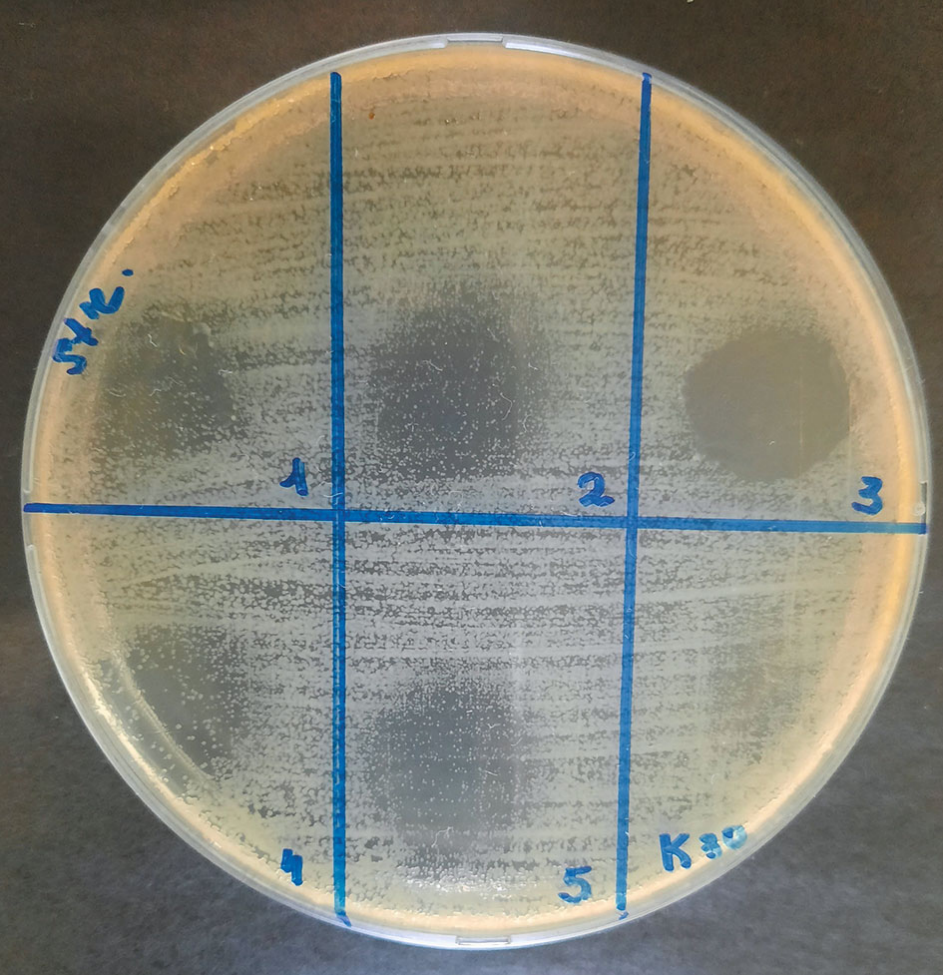
Effect of some lipid extracts of *Culcita novaeguineae* on *S. pyogenes*.

**Fig. 2.**
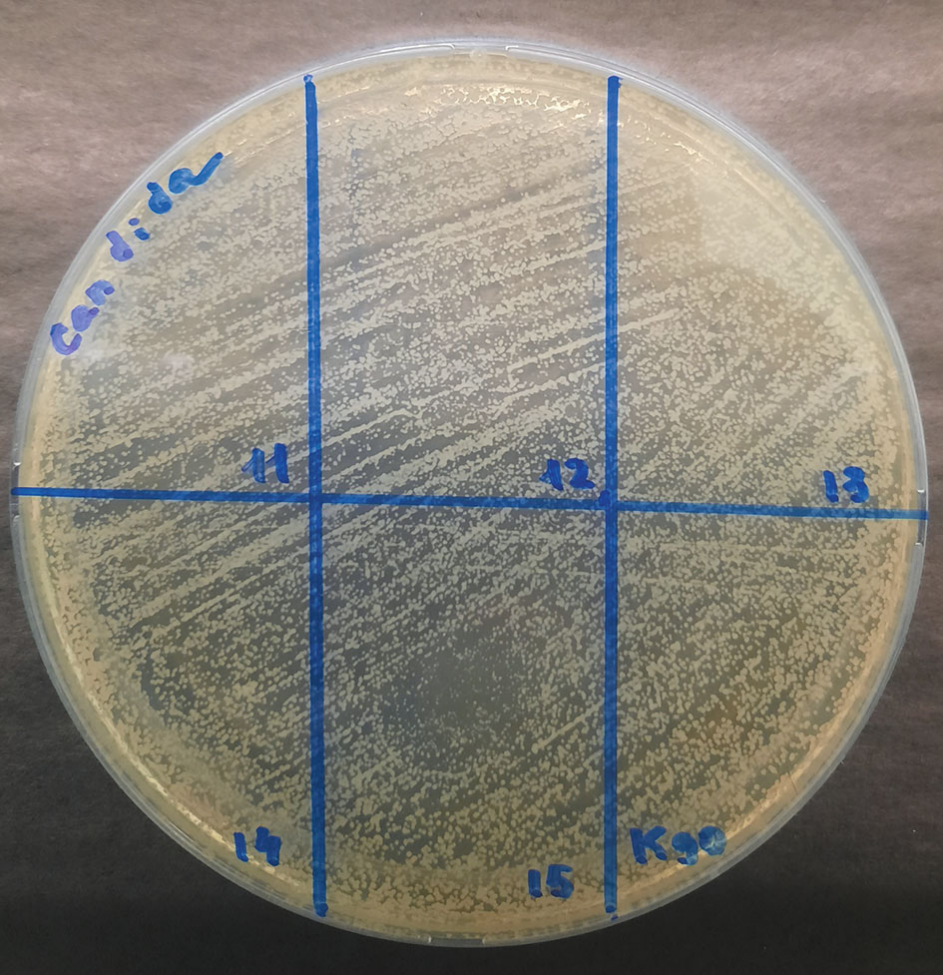
Effect of some lipid extracts of *Culcita novaeguineae* on *Candida* sp.

It was established that lipid extracts of echinoderms from Nha Trang Bay exhibit the greatest antimicrobial activity against *S. pyogenes* (Figs. 3–5). For example, Fig. 3 shows that the lipid extracts of the starfish *C. novaeguineae* sample no. 81 (together with DMSO at a concentration of 30%) and sample nos. 91 and 93 (DMSO concentration 90%) showed a complete bactericidal effect against *S. pyogenes*, whereas a bacteriostatic effect of 30% DMSO solution and an incomplete bactericidal effect of a 90% DMSO solution were observed in the control. The effect of extracts of echinoderms *D. setosum* and *L. laevigata* at a DMSO concentration of 30% on the studied microorganism varied from incomplete to complete bactericidal effect, whereas either the absence of effect or a bacteriostatic effect of the samples was recorded in the control (Figs. 4, 5). A number of *D. setosum* extracts at DMSO concentrations of 30 and 50% had a bacteriostatic effect on isolates of *S. aureus* and *Candida* sp. in the absence of effect in control samples (Fig. 4). When studying the antibacterial effect of lipid extracts on the Gram-negative microorganisms *K. pneumoniae*, *E. coli*, and *P. aeruginosa*, a bacteriostatic and incomplete bactericidal effect of some extracts of *C. novaeguineae* at a concentration of DMSO of 90% was observed, whereas the effect in the control samples was absent (Fig. 3). Extracts of *D. setosum* had a bacteriostatic effect on *P. aeruginosa* and *E. coli* at a DMSO concentration of 90% (Fig. 4); a similar effect on *P. aeruginosa* was found for lipid extracts of the starfish *L. laevigata* (Fig. 5). The lipid extracts of *L. laevigata*, *D. setosum*, and *C. novaeguineae* showed no antimicrobial activity against *E. faecium* (Figs. 3–5).

**Fig. 3.**
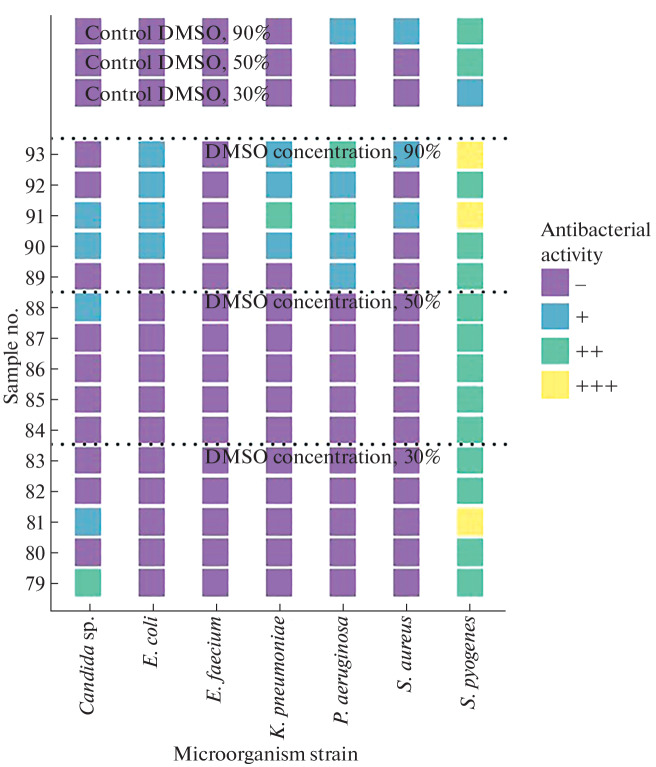
Antimicrobial activity of lipid extracts of *Culcita novaeguineae*.

**Fig. 4.**
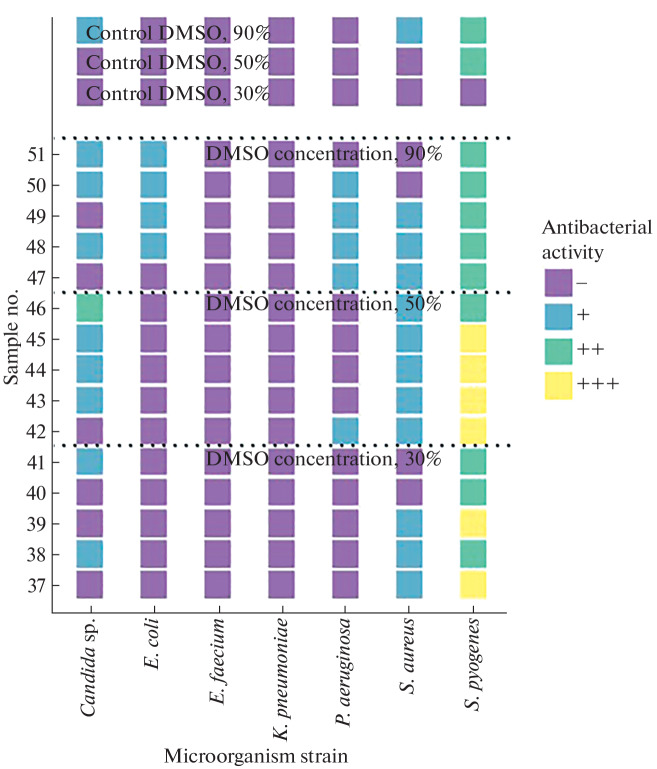
Antimicrobial activity of lipid extracts of *Diadema setosum*.

**Fig. 5.**
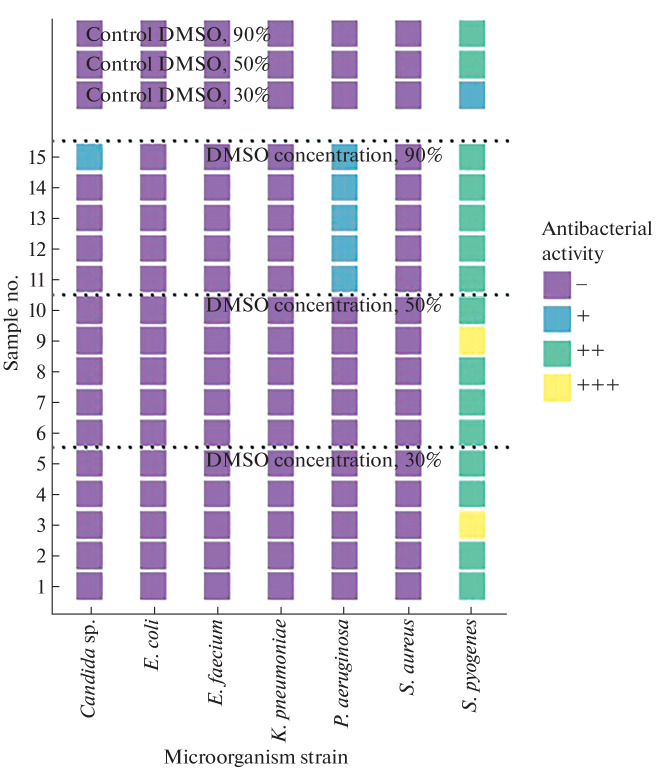
Antimicrobial activity of lipid extracts of *Linckia laevigata*.

The studied echinoderm species differed in the content of total lipids (TLs) and in the quantitative composition of lipid classes (Table 2). For example, the largest amount of TLs was shown for the sea urchin *D. setosum* (25% of dry matter) mainly due to nonpolar lipids, triacylglycerols (5.87%), which practically do not exhibit antimicrobial activity. For the starfishes *L. laevigata* and *C. novaeguineae*, the TL content was 14.2 and 13.8%, respectively. The dominant classes of lipids in starfishes were free fatty acids, phospholipids, and cholesterol, the antimicrobial properties of which are quite pronounced. For example, free fatty acids are involved in metabolic processes that lead to the suppression of signal transmission from cell to cell, preventing cell adhesion and the formation of a bacterial film [9]. The fatty acid profile of total lipids of starfishes was characterized by the dominance of polyunsaturated fatty acids, PUFAs (45% of the total fatty acids in *C. novaeguineae* and 52% in *L. laevigata*) due to the prevalence of n-6PUFAs (20 and 16%, respectively). Among the latter, arachidonic fatty acid 20:4 (n-6) dominated. The content of monounsaturated fatty acids, MUFAs, varied from 30 to 34% due to 20:1 (n-11), 18:1 (n-9), and 18:1 (n-7). The content of saturated fatty acids, SFAs, ranged from 18 to 21% with the dominance of 18:0, 16:0, and 20:0 FAs due to 16:0 and 14:0 (24 and 8%, respectively). The next in quantitative respect were PUFAs (31.5%), in which n-6PUFAs accounted for 18% due to 20:4 (n-6), and the content of MUFAs was 26% due to the dominance of 16:1 (n-7) (Table 3). There are data that unsaturated FAs are more active against the Gram-positive bacteria and that the antimicrobial properties of FAs increase with an increase in their degree of unsaturation and are associated with the position of the double bond [10]. It should be noted that the pronounced antimicrobial properties of arachidonic acid and its derivatives (eicosanoids), as well as eicosapentaenoic and docosagensaenoic acids isolated from different sources are well known, and the mechanism of their action is wide and multifactorial [11].

**Table 2.** Content of total lipids and lipid classes (phospholipids, triacylglycerols, diacylglycerols, cholesterol esters, cholesterol, free fatty acids) (% dry weight) in the studied tissues of some echinoderm species (*Linckia laevigata*, *Diadema setosum*, *Culcita novaeguineae*)

Species	*Linckia laevigata*	*Diadema setosum*	*Culcita novaeguineae*
n	15	15	15
TLs	14.20 ± 0.90	24.98 ± 1.02	13.80 ± 0.53
PLs	3.26 ± 0.26	3.38 ± 0.21	3.10 ± 0.12
MAGs	1.35 ± 0.11	1.42 ± 0.14	1.36 ± 0.04
DAGs	0.40 ± 0.03	3.18 ± 0.26	0.51 ± 0.06
Chol	3.37 ± 0.24	4.21 ± 0.23	3.53 ± 0.10
FFAs	3.80 ± 0.31	5.53 ± 0.46	3.26 ± 0.23
TAGs	0.31 ± 0.09	5.87 ± 1.12	0.75 ± 0.15
Chol esters	1.70 ± 0.14	1.38 ± 0.19	1.29 ± 0.05

TL—total lipids, PL—phospholipids, MAGs—monoacylglycerols, DAGs—diacylglycerols, Chol—cholesterol, FFA—free fatty acids, TAGs—triacylgrlycerols, Chol esters—cholesterol esters.

**Table 3.** Fatty acid composition (% of the total fatty acids) in the studied tissues of some echinoderm species (*Linckia laevigata*, *Diadema setosum*, *Culcita novaeguineae*)

Species	*Linckia laevigata*	*Diadema setosum*	*Culcita novaeguineae*
n	5	5	5
14:0	0.65 ± 0.09	8.43 ± 1.10	0.46 ± 0.04
16:0	3.82 ± 0.16	24.33 ± 0.69	4.30 ± 0.64
17:0	1.05 ± 0.07	0.95 ± 0.14	0.87 ± 0.07
18:0	8.27 ± 0.13	5.02 ± 0.13	10.10 ± 1.04
20:0	2.67 ± 0.27	1.93 ± 0.10	3.37 ± 0.29
∑ SFA	18.01 ± 0.30	42.12 ± 1.03	21.25 ± 2.15
18:1 (n-9)	8.72 ± 0.47	3.56 ± 0.78	5.23 ± 0.72
18:1 (n-7)	3.46 ± 0.07	3.61 ± 0.16	4.49 ± 0.47
20:1 (n-11)	10.49 ± 0.39	3.08 ± 0.21	10.73 ± 0.43
20:1 (n-9)	0.62 ± 0.06	3.62 ± 0.22	1.19 ± 0.16
∑ MUFA	29.98 ± 0.72	26.35 ± 0.72	33.67 ± 3.20
14:2 (n-9)	0.65 ± 0.09	0.47 ± 0.05	0.30 ± 0.04
16:2 (n-9)	0.73 ± 0.05	0.36 ± 0.02	0.40 ± 0.07
18:2 (n-9)	0	0	1.27 ± 0.31
20:3 (n-9)	6.16 ± 0.51	0.14 ± 0.03	0.23 ± 0.04
∑ (n-9) PUFA	8.03 ± 0.42	1.32 ± 0.07	2.61 ± 0.50
18:2 (n-7)	4.15 ± 0.22	0.06 ± 0.01	0.39 ± 0.14
∑ (n-7) PUFA	4.95 ± 0.22	0.36 ± 0.01	0.97 ± 0.20
18:2 (n-6)	1.10 ± 0.10	1.79 ± 0.12	0.67 ± 0.08
20:4 (n-6)	12.93 ± 0.88	12.28 ± 0.29	17.90 ± 1.46
∑ (n-6) PUFA	15.91 ± 0.90	18.10 ± 0.63	19.98 ± 1.37
18:2 (n-4)	6.68 ± 0.39	0.11 ± 0.02	0.70 ± 0.21
18:3 (n-4)	1.52 ± 0.69	0.04 ± 0.02	2.21 ± 0.30
∑ (n-4) PUFA	10.45 ± 0.89	0.86 ± 0.13	6.24 ± 0.51
18:3 (n-3)	0	0.89 ± 0.08	0.73 ± 0.27
18:4 (n-3)	2.48 ± 0.20	1.73 ± 0.17	0
20:5 (n-3)	6.24 ± 0.15	5.01 ± 0.30	8.76 ± 0.70
22:5 (n-3)	0.45 ± 0.21	0.27 ± 0.02	0.88 ± 0.54
22:6 (n-3)	0.83 ± 0.04	1.25 ± 0.05	1.35 ± 0.51
∑ (n-3) PUFA	12.67 ± 0.19	10.85 ± 0.51	14.78 ± 0.97
∑ PUFA	52.01 ± 0.53	31.5 ± 0.73	45.08 ± 1.23

The table shows individual fatty acids that dominate the FA family or account for more than 1%. SFAs are saturated fatty acids, MUFAs are monounsaturated fatty acids, and PUFAs are polyunsaturated fatty acids.

The results of the studies indicate that the lipid extracts of echinoderms from Nha Trang Bay exhibit the highest antimicrobial activity against the Gram-positive bacteria, namely, *S. pyogenes*.

It was shown that the studied hydrobionts differed both in qualitative and quantitative composition of lipids and fatty acids, while exhibiting a similar antimicrobial effect. For example, the lipid profile of starfishes was similar and was characterized by the exclusive dominance of PLs, Chol, and FFAs; Chol esters and MAGs ranked second; and TAGs and DAGs were minor. A different composition of lipids was found in the sea urchin, which was characterized by the dominance of TAGs and FFAs, followed by Chol, then total PLs, whereas MAGs and Chol esters were minor. Quantitative differences in the content of the studied lipids in the sea urchin are more even as compared to starfishes: the amount of PLs, Chol, and FFAs was 2.8 times higher than that of Chol esters and MAGs. The established antimicrobial activity of the studied starfish species is probably due to the increased content of free fatty acids and phospholipids, as well as PUFAs (from 45 to 52% of the total FAs), in particular, omega-6 PUFAs, which dominated the FA profile of these hydrobionts.

Interestingly, when studying the antibacterial effect of sea urchin extracts, their effect on *S. pyogenes* was established. TAGs and metabolically labile FFAs were the dominant classes of lipids, their content exceeded that in starfishes. The fatty acid profile of total lipids was characterized by the dominance of FFAs (42% of the total FAs) due to palmitic acid, 16:0, whose antimicrobial properties are widely known [12], followed by a significant amount of PUFAs (31.5% of the total FAs), which were also dominated by (n-6) PUFAs.

According to modern ideas, PUFAs have a significant antimicrobial effect on many organisms, including bacteria, viruses, fungi, and some parasites. At the same time, there is evidence that PUFAs also have an immunomodulatory effect on the human body and are able to enhance both cellular and humoral immune responses [11].

Free fatty acids are also involved in the formation of antitumor immunity by increasing the production of free radicals in tumor cells [13]. In addition, other studies have shown that fatty acids have anti-inflammatory and wound-healing effects [14–16].

The antibacterial and immunomodulatory properties of PUFAs contained in echinoderm lipid extracts make them promising objects for creating antimicrobial agents for the treatment of infections of various localizations. A likely method of using such agents will be the external treatment due to the possible processes of biotransformation of extracts during injection or oral administration. In the study by L.Yu. Lazhen-tseva, the results of clinical trials of a new antimicrobial drug made from marine fish lipids as an external antiseptic in the complex therapy of local purulent-inflammatory diseases of the skin, soft tissues, and mucous membranes are presented, and the effectiveness of the drug in purulent-inflammatory wound processes, urogenital diseases, and microbial eczema is shown [17].

The results of the study of the antimicrobial activity of echinoderms from Nha Trang Bay are of interest in terms of studying the mechanisms of action of these extracts, as well as the search for various compositions of lipids and their fatty acid components that exert an antibacterial effect, including that against the antibiotic-resistant strains of microorganisms.
